# Beyond visual comfort: comparing pupillometry and subjective measures of cognitive strain in workplace lighting

**DOI:** 10.3389/fpubh.2026.1875100

**Published:** 2026-09-03

**Authors:** D-Filipa Ferreira, Ana-Carolina Fonseca, Simão Ferreira, Luís Pinto-Coelho, Matilde A. Rodrigues

**Affiliations:** 1 RISE-Health, Center for Translational Health and Medical Biotechnology Research (TBIO), ESS, Polytechnic of Porto, Porto, Portugal; 2 ALGORITMI Research Centre/LASI, University of Minho, Guimarães, Portugal; 3 INESCTEC, Campus of the Engineering Faculty of the University of Porto, Porto, Portugal; 4 ISEP, Polytechnic of Porto, Porto, Portugal

**Keywords:** cognitive load, ergonomics, illuminance, occupational health, screen-based tasks

## Abstract

**Introduction:**

This study evaluated the discriminative sensitivity of pupillometry, relative to subjective self-report measures, as an objective indicator of cognitive load during screen-based tasks performed under two workplace illuminance levels (300 and 500 lux). Inadequate workplace illuminance is associated with visual fatigue, stress, and impaired attention, yet its effect on cognitive strain during screen-based tasks remains underexplored through objective physiological indicators, particularly given the confounding influence of the pupillary light reflex (PLR) on pupillometric measures.

**Methods:**

Forty-eight working-age adults performed writing and working memory (N-back) tasks under both conditions in a controlled laboratory replicating office environments; pupil diameter was recorded continuously using Pupil Labs Core eye-tracking glasses, while the NASA-TLX and Visual Analog Scales (VAS) assessed subjective workload, stress, fatigue, and eye strain.

**Results:**

A direct comparison between illuminance conditions (Holm-corrected) revealed no significant effects of illuminance on pupillary parameters (|d| ≤ 0.19), but subjective workload increased with illuminance during the most demanding task (*d* = 0.81). Both pupillometry and subjective measures detected significant pairwise differences between task demand levels consistently under both illuminance conditions (Kendall's W ranging from 0.29–0.35 for pupil diameter variance to 0.34–0.58 for NASA-TLX and VAS Stress). No significant differences were found for the writing task or the 2-back task across any measure.

**Discussion:**

The results indicate that pupillometry and subjective self-report measures are comparably sensitive to task demand, while only subjective workload was sensitive to illuminance, underscoring the value of combining objective and subjective indicators in occupational lighting research.

## Highlights

- Pupillometry did not find significant differences between 500 lux and 300 lux, while NASA-TLX showed higher perceived workload at 500 lux for the most demanding task.
- Subjective and objective measures diverged systematically, with participants reporting varied perceptions of fatigue and comfort that did not align with physiological indicators.
- Both pupillometry and subjective measures (NASA-TLX, VAS) showed consistent task-level sensitivity, detecting significant differences between task demand levels across both illuminance conditions.
- The study highlights the complementary value of combining objective physiological metrics with self-report in occupational health assessments of workplace lighting.
- Methodological limitations, including fixed task order and absent screen contrast data, are acknowledged; their implications for causal attribution are discussed.

## 1 Introduction

Cognitive demands in screen-intensive work environments have become a critical focus in human factors and ergonomics, with documented consequences for both performance and worker wellbeing (1, 2). Mental workload, the relationship between a task's cognitive demands and an individual's available information-processing capacity, is central to this domain (3). Evidence indicates that cognitive overload, fatigue, and cognitive strain substantially increase the risk of errors and workplace accidents (4–6). Among the environmental factors that influence this, lighting is particularly salient: low illuminance has been associated with visual fatigue, stress, and attentional difficulties, each capable of compromising performance and safety (7–9).

Although these terms are related, they refer to distinct constructs. Cognitive load refers to the processing demands imposed on working memory by a task or learning material (10). Mental workload is the broader relationship between a task's cognitive demands and an individual's available information-processing capacity, encompassing cognitive, temporal, and effort-related components (3). Cognitive strain, for which there is no single standardized definition in the literature, is operationally defined as the physiological and subjective consequences of sustained or excessive workload, including fatigue, stress, and reduced performance (4, 6). This study draws on indicators of cognitive load (pupillometry) and mental workload (National Aeronautics and Space Administration Task Load Index – NASA-TLX) to characterize cognitive strain as the integrated subjective and physiological response to sustained cognitive demands.

Despite international occupational standards recommending a minimum of 500 lux for office environments (EN 12464-1), real-world workplaces frequently fall short of this threshold (11–14). It should be noted that lighting guidance is not fully uniform on this point: while EN 12464-1 recommends 500 lux for general office tasks including keyboard and data-entry work, other guidance (e.g., SLL/CIBSE Lighting Guide 7) recommends 300 lux for predominantly screen-based tasks and reserves 500 lux for paper-based work. This divergence reflects the fact that emissive displays are less dependent on ambient illuminance for visibility than reflective media and complicates a simple characterization of 300 lux as universally ‘inadequate' for screen-based work—a nuance the present study's findings help to inform. Importantly, most research and standards focus predominantly on visual comfort and task performance using reflective display media (e.g., paper). The relationship between illuminance and cognitive load in screen-based tasks, where emissive displays introduce an additional interaction with ambient light through contrast modulation, remains comparatively underexplored, particularly through objective psychophysiological indicators (7, 15).

Pupillometry is a promising tool for this purpose. Changes in pupil diameter provide a real-time, non-invasive proxy of cognitive effort (16–21). However, its application in lighting research is complicated by the pupillary light reflex (PLR): ambient illuminance directly drives constriction, creating a confound when pupil size is used as an index of cognitive state under varying light levels (22, 23). This dual sensitivity, to both light and cognitive load, raises important methodological questions that warrant explicit consideration.

The present study uses Pupil Labs Core (PLC) eye-tracking technology (24) to examine whether two illuminance levels (300 and 500 lux) produce measurable differences in pupillary dynamics during screen-based cognitive tasks, and whether these differences align with or diverge from subjective assessments (NASA-TLX and VAS scales). Rather than treating pupillometry as a straightforward measure of workload under varying light, this study specifically investigates its sensitivity and discriminative capacity relative to self-report, contributing to a methodological evidence base for occupational health assessment.

Building on this rationale, the relationship between mental workload and cognitive overload (3, 4), the gap between illuminance standards and screen-based research (7, 11, 15), and the PLR confound in pupillometric measurement (22, 23), the following research question was formalized. The PICO framework was used to define the research question: *How do different workplace illuminance levels affect cognitive performance factors in screen-intensive tasks, and to what extent can pupillometry complement or diverge from subjective assessment methods in characterizing these effects?* Components: *P* — Workers in computer-intensive roles (≥3 h/day screen-based tasks); I — Exposure to illuminance levels of 300 vs. 500 lux; C — Differences in cognitive performance indicators between conditions assessed through pupillometry and standardized questionnaires; O — Characterization of illuminance effects on cognitive strain, and evaluation of pupillometry as a complementary occupational assessment tool.

## 2 Materials and methods

This study adopted an analytical experimental design to evaluate how workplace illuminance affects indicators of cognitive performance. Specifically, it investigated whether 300 and 500 lux conditions produce different patterns in pupil diameter metrics and subjective ratings of workload, stress, fatigue, and eye strain.

### 2.1 Participants

Working-age adults regularly engaged in screen-based tasks (>3 h/day) were recruited via convenience sampling. No ocular exclusions were applied. Ethics approval was obtained from the ethics committee of the Polytechnic of Porto School of Health (CE0041E). All participants provided informed consent in accordance with the Declaration of Helsinki.

Of 90 invited, 51 consented and 48 completed the protocol (41.7% male, *n* = 20; 58.3% female, *n* = 28; mean age 24 years). A pre-task baseline questionnaire (LimeSurvey – an online survey platform; English/Portuguese) captured initial psychological state and potential confounders, including the Depression, Anxiety and Stress Scale (DASS-21) (25, 26), Penn State Worry Questionnaire (PSWQ) (27, 28), and Coping Orientation to Problems Experienced Inventory (Brief-COPE) (29, 30). Additional participant characteristics included corrective lens use, facial hair, make-up, Fitzpatrick skin type, body mass index (BMI), mental health history, and substance use. These variables were collected to identify factors that could affect eye-tracking quality (e.g., corrective lenses, facial hair, and make-up), pupil detection accuracy (e.g., Fitzpatrick skin type), or physiological pupillary responses (e.g., BMI, mental health history, and substance use), enabling their potential influence on the results to be evaluated.

### 2.2 Experimental conditions and illuminance levels

**Figure 1:**
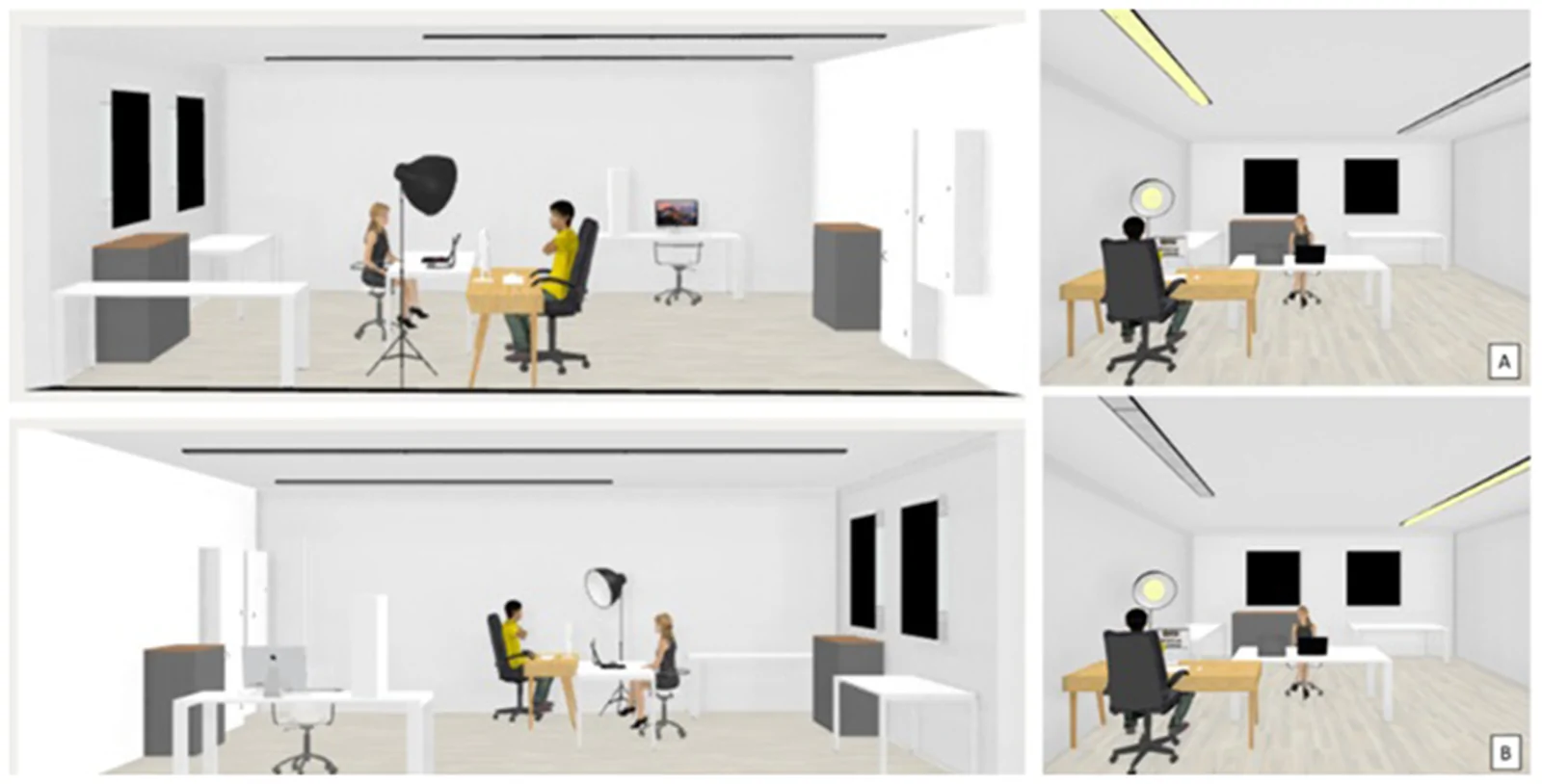
On the left: Left and Right side views of the simulated working environment setup showing the participant (in yellow) and the experimenter (in black), with the luminaire arrangement. Note that it is not in scale, its representative, no light was directly pointed to the participants face. On the right: 3D model of the Human Factors & Collaboration room lighting conditions; **(A)** 500 lux; **(B)** 300 lux.

The Human Factors & Collaboration room was configured to simulate a standard office environment. Illuminance levels were set to 500 lux and 300 lux (EN 12464-1), with condition order randomized per participant. A matte, non-reflective desk surface and controlled screen positioning were used to minimize veiling reflections and reflected glare, in line with workplace-lighting guidance. The protocol used was 60 cm viewing distance, screen-source angles designed to minimize specular reflections, and the floor luminaire was positioned approximately 2 m from the participant at 45° (31). Seating position was standardized in terms of its location within the room (fixed viewing distance and screen-source geometry); chair height and backrest were adjusted individually for each participant's ergonomic comfort, which may have introduced minor inter-individual variation in eye height and gaze direction relative to the light source. The experiment was conducted without daylight contribution, the light conditions are displayed in Figure 1B, two set of ceiling luminaires existed, the one above the participant was on to get 500 lux, and off on 300 lux, the other one (participants left) was reverse on and off.

It should be noted that while glare and screen contrast were minimized by design (matte surfaces, controlled angles), screen contrast ratio under each illuminance condition was not formally measured. This is because the research team did not have access to the luminance probe required for this measurement; this is acknowledged as an equipment limitation in the Discussion rather than a deliberate omission. Future studies should characterize the luminance of the screen surface under each ambient condition to rule out contrast-mediated effects on task difficulty. ENLIGHT checklist (32) is provided in Supplementary Appendix 1.

Environmental parameters were monitored with standardized equipment throughout each session (Table 1). Ocular illuminance was assessed three times at the level of each eye immediately after completion of the tasks under each lighting condition. Additionally, ambient illuminance was monitored during task performance at six measurement points within the task area and three points within the neighborhood area to ensure that the target illuminance levels were maintained consistently throughout the experiment. Measured task-area illuminance was 511 ± 24 lux (500 lux condition) and 270 ± 12 lux (300 lux condition), with uniform ocular illuminance confirmed for both. Mean noise was 45 ± 2 dB. Temperature (≈24 °C), relative humidity (≈56%), and color temperature (≈3710 K) were stable across conditions.

**Table 1:** Environmental variables monitored during experimental sessions.

Measurand/variable	Unit	Equipment type	Brand/model
Air temperature	°C	Thermohygrometer	VelociCalc multifunctional 9565-X, TSI
Air relative humidity	%	Thermohygrometer	VelociCalc multifunctional 9565-X, TSI
Task, neighbourhood and ocular illuminance	lux	Luxmeter	Delta-OHM, HD 2302.0; LP 471 phot probe
Noise	dB	Dosimeter	Soundmeter, Bruel & Kjaer 2250-D00
Colour temperature	Kelvin	Spectroradiometer	Gossen-Mavospec base spectral

### 2.3 Assessment instruments

#### 2.3.1 Subjective measures

Subjective workload was assessed using the NASA-TLX (33), which evaluates six dimensions (mental demand, physical demand, temporal demand, performance, effort, and frustration) via 100-point scales. Raw scores were calculated using the standard unweighted averaging method.

Perceived stress was assessed via a 100 mm Visual Analogue Scale (VAS), anchored at 0 (“not at all”) and 100 (“a great deal”), administered after each individual task, whereas fatigue and eye strain were assessed via VAS administered once per lighting-condition block (not after each individual task).

#### 2.3.2 Objective measure: pupillometry

Pupil diameter was recorded continuously using PLC eye-tracking glasses throughout all experimental tasks. All participants performed an initial calibration procedure, which allows the system to report absolute pupil diameter values (in mm). Raw pupil data were cleaned using a 2–8 mm physiological plausibility filter (34), segmented by task using time-based boundaries, and averaged across both eyes. Descriptive metrics (mean, amplitude, variance, minimum and maximum values) were calculated from the cleaned pupil diameter time series. Amplitude was defined, for each task and condition, as the difference between the maximum and minimum pupil diameter recorded within that segment (max – min), and is therefore distinct from the raw maximum value itself.

### 2.4 Protocol

After completing the baseline questionnaire, participants performed cognitive tasks under both illuminance conditions (order randomized per participant). Each block comprised a 2-back task, a 3-back task, and a writing task. The writing task required participants to compose an email based on a written prompt presented on the screen. Different but comparable prompts were used in each illuminance condition (e.g., requesting annual leave or writing to reschedule a meeting) to reduce learning effects between repeated assessments. After each N-back block and the writing task, participants completed the NASA-TLX. Between illuminance conditions, an approximately 2 min adaptation period was allowed. Pupil diameter was recorded continuously across all tasks. Each participant completed the full protocol, including both illuminance conditions, in a single session lasting approximately 30 min, 15 for each condition. The duration of the N-back tests varied across participants due to differences in response speed. In the writing task, participants had a maximum of 2 min to complete the task; however, no minimum time was imposed, resulting in variability in completion times across participants.

**Figure 2:**
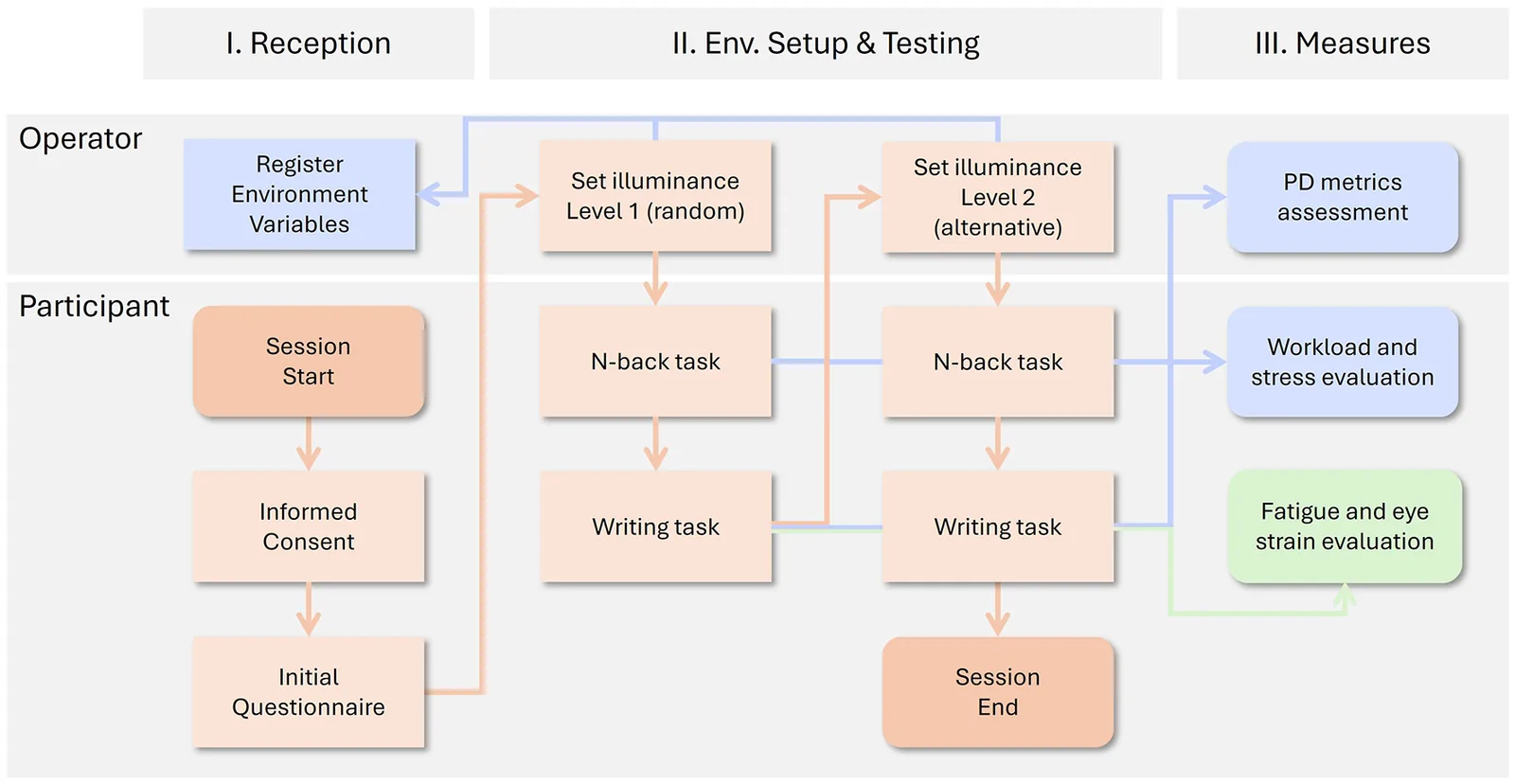
Pipeline for the assessment of cognitive performance factors. PD, Pupil diameter.

Task order within each condition was fixed: 2-back → 3-back → writing task (Figure 2). This was not counterbalanced across participants. The implications of this sequential design for the interpretation of pupillary data are discussed in the Limitations section.

N-back accuracy data (percentage of correct responses) were excluded from analysis due to a technical artifact: insufficient keypress force on the spacebar caused unregistered responses for a subset of participants (*n* = 12–14 per condition), producing a bimodal distribution (0% accuracy in affected participants; >80% in unaffected participants). Post-experiment debriefing confirmed this was a peripheral input device issue unrelated to task performance or illuminance condition. The potential influence of this artifact on pupillometric indicators is assessed empirically in Section 3.2.

### 2.5 Data analysis

All analyses were conducted in Python 3.9 (Jupyter Notebooks). Prior to all comparisons, pupil data were normalized on a per-participant basis using global min–max scaling, i.e., (X-min_participant_)/(max_participant_ – min_participant_), where the minimum and maximum were extracted from each participant's entire pupil time series across all conditions. This procedure was applied to control for interindividual differences in baseline pupil diameter and physiological range. Subjective measure data were also normalized to a 0–1 range: since the original scales were 0–100, this was achieved by simple division by 100. For other questionnaire-based measures, standard min–max normalization was applied, i.e., (X-min)/(max – min). Two distinct sets of comparisons were then performed, corresponding to two separate research questions. First, differences across tasks (2-back, 3-back, writing) within each illuminance level were examined using Friedman test with Nemenyi *post-hoc* comparisons, consistent with the normality results. Second, and independently of the within-condition task comparisons, the same metrics were compared between the two illuminance conditions (300 vs. 500 lux) for each task separately, using paired-samples *t*-tests or Wilcoxon signed-rank tests depending on normality, since these measurements represent repeated assessments from the same participants under both lighting conditions. Normality for paired comparisons was assessed using the Shapiro–Wilk test on the difference scores (X500–X300) for each task and metric, as this is the appropriate distributional assumption for paired tests. Correlations between pupil diameter metrics and baseline psychological measures were assessed using Pearson or Spearman coefficients as appropriate, here normality of normalized data was assessed using the Shapiro-Wilk test (see Supplementary Appendix 2 for normality results per metric).

## 3 Results

### 3.1 Sample characterisation

**Figure 3:**
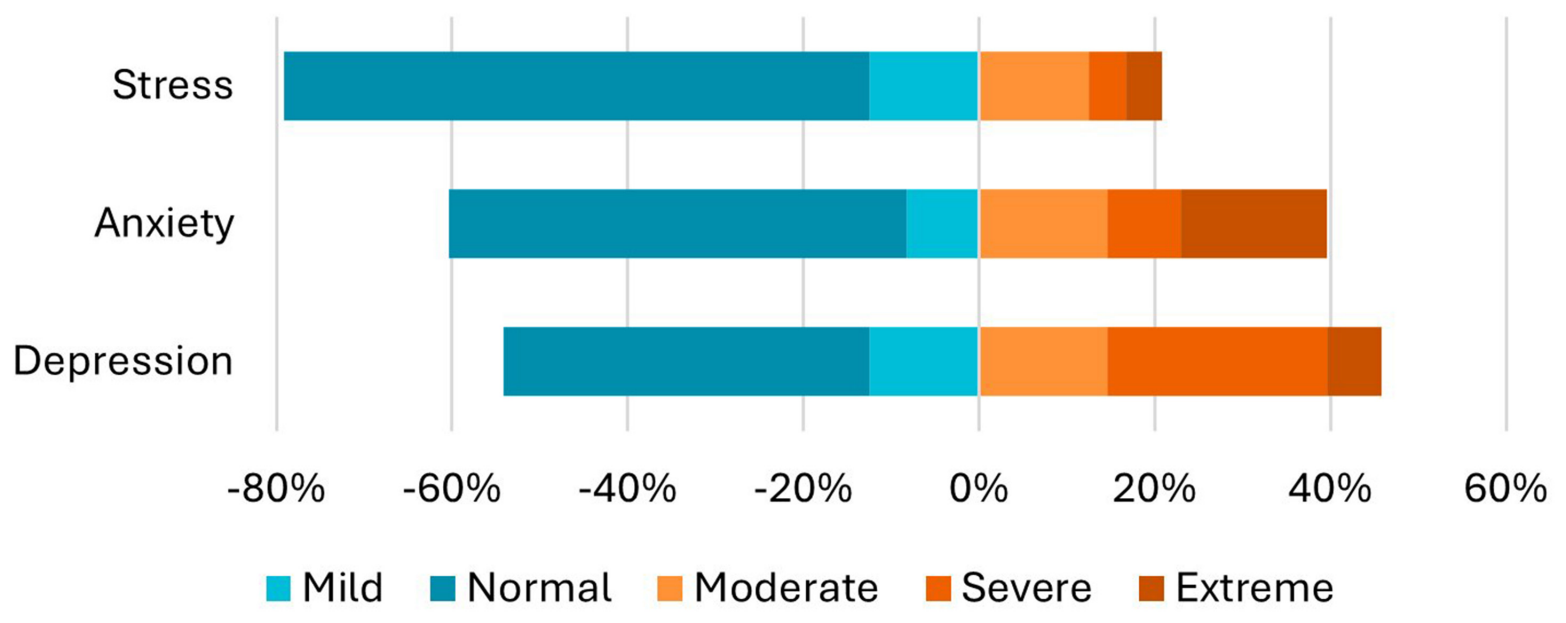
DASS-21 scores participants distribution.

The final sample comprised 48 participants (41.7% male (*n* = 20), 58.3% female (*n* = 28); mean age 24 years). Most participants (68.75%) reported problem-focused coping as their primary strategy, and worry levels were predominantly moderate (66.67%). DASS-21 scores showed a predominance of normal-range values for depression, anxiety, and stress, with few extreme cases (Figure 3).

Significant negative correlations were observed between age and mean pupil diameter, consistent with the known age-related decline in pupil reactivity (35). Age was also associated with greater use of both problem-focused (*r* = 0.30, *p* < 0.05) and emotion-focused (*r* = 0.41, *p* < 0.01) coping strategies.

Regarding pupil diameter and psychological state, for the DASS-21 subscales, several significant associations emerged. Pupil variance during the writing task under 300 lux showed negative correlations with Depression (ρ = −0.369, *p* = 0.010) and Stress (ρ = −0.317, *p* = 0.028). Similarly, pupil amplitude during the same task and lighting condition was also negatively correlated with Depression (ρ = −0.406, *p* = 0.004). In contrast, pupil mean diameter during the 2-back task at 500 lux was positively correlated with Depression (ρ = 0.335, *p* = 0.020), while mean diameter during the writing task at 500 lux was positively correlated with both Anxiety (ρ = 0.444, *p* = 0.002) and Worry/Preoccupation (ρ = 0.319, *p* = 0.027). Additionally, mean diameter during the 3-back task at 300 lux showed a positive correlation with Anxiety (ρ = 0.331, *p* = 0.021). No significant correlations were found between maximum pupil diameter and any of the DASS-21 subscales (all ps > 0.05). Regarding coping methods, pupil amplitude during the writing task at 500 lux was positively correlated with problem focused score (ρ = 0.307, *p* = 0.034), and pupil maximum during the 2-back task at 500 lux showed a negative correlation with emotional focused score (ρ = −0.379, *p* = 0.008). No significant correlations were found with avoidant scores across any metric or condition. No significant effect of facial features (glasses, make-up, beard) on PLC calibration was observed.

These correlational findings are exploratory and should not be interpreted as establishing causal relationships between psychological state and pupil diameter in this experimental context. A fully powered study specifically designed to examine individual differences would be required to draw stronger conclusions.

### 3.2 Pupillometric results

Descriptive statistics for pupil diameter metrics under each condition and task are presented in Table 2.

**Table 2:** Mean descriptive statistics for pupil diameter (mm) by illuminance condition and task.

Condition	Task	Mean (mm)	Amplitude (mm)	Variance	Min (mm)	Max (mm)
500 lux	2-back	3.71	2.70	0.27	2.00	7.94
	3-back	3.36	1.56	0.16	2.00	7.47
	Writing	3.73	1.88	0.22	2.01	7.55
300 lux	2-back	3.62	2.72	0.28	2.97	8.00
	3-back	3.36	1.62	0.14	2.97	6.54
	Writing	3.61	1.96	0.23	2.97	7.09

Pupil diameter variance differed significantly across tasks under both 500 lux (Friedman χ^2^ = 27.875, *p* < .001, Kendall's W: 0.290) and 300 lux (χ^2^ = 33.5, *p* < 0.001, Kendall's W: 0.349). The larger Friedman statistic under 300 lux indicates that tasks were more reliably differentiated in that condition; it does not indicate higher variance *per se*, which is instead addressed directly in Table 3. The proportion of participants showing pupil constriction (defined as a decrease in mean diameter from the first 5 s to the last 5 s of the session) was 79.17% under 300 lux and 75.00% under 500 lux. A chi-square test of independence showed no significant difference between the two conditions (χ^2^ = 0.059, df = 1, *p* = 0.808). Adittionally, participants showed higher pupil diameter amplitudes alongside smaller mean diameters under 300 lux.

**Table 3:** Results of direct comparison between illuminance conditions (500 vs. 300 lux) for each task with normalised data. WT, writing task.

Metric	Task	Normality *p*	Test used	Statistic	*p* (uncorrected)	*p* (Holm)	Effect size
PD variance	2-back	0.160	paired *t*-test	−1.31	0.20	0.59	−0.19
	3-back	0.000	Wilcoxon	571.00	0.87	0.87	0.02
	WT	0.020	Wilcoxon	474.00	0.25	0.59	0.17
PD amplitude	2-back	0.697	paired *t*-test	−0.37	0.72	1.00	−0.05
	3-back	0.065	paired *t*-test	−0.22	0.83	1.00	−0.03
	WT	0.670	paired *t*-test	−1.26	0.21	0.64	−0.18
PD mean	2-back	0.388	paired *t*-test	0.57	0.57	1.00	0.08
	3-back	0.035	Wilcoxon	561.00	0.79	1.00	0.04
	WT	0.692	paired *t*-test	1.08	0.29	0.86	0.16
PD maximum	2-back	0.030	Wilcoxon	509.00	0.42	1.00	0.12
	3-back	0.603	paired *t*-test	0.20	0.84	1.00	0.03
	WT	0.599	paired *t*-test	−0.37	0.71	1.00	−0.05
PD minimum	2-back	0.374	paired *t*-test	0.42	0.68	0.68	0.06
	3-back	0.409	paired *t*-test	1.04	0.30	0.61	0.15
	WT	0.164	paired *t*-test	1.29	0.20	0.61	0.19
NASA-TLX	2-back	0.000	Wilcoxon	518.00	0.48	0.96	0.10
	3-back	0.748	paired *t*-test	5.58	0.00	0.00	0.81
	WT	0.834	paired *t*-test	0.33	0.74	0.96	0.05
VAS Stress	2-back	0.375	paired *t*-test	−0.55	0.58	1.00	−0.08
	3-back	0.148	paired *t*-test	0.05	0.96	1.00	0.01
	WT	0.016	Wilcoxon	436.00	0.85	1.00	0.03

Direct pairwise comparisons on normalized data between illuminance conditions (500 vs. 300 lux), with Holm correction for multiple comparisons, were performed on all normalized pupil diameter metrics. The results consistently showed that ambient illuminance did not significantly modulate any pupillary metric, including variance, amplitude, mean, maximum, and minimum, across any of the three tasks (2-back, 3-back, and writing). All adjusted *p*-values exceeded the 0.05 threshold, and effect sizes were uniformly small (|d| ≤ 0.19), confirming the absence of a reliable effect of lighting condition on pupillary responses under the present experimental setup.

To rule out the possibility that these differences were driven by truncation at the pupil diameter plausibility filter boundaries (2–8 mm), a sensitivity analysis was performed excluding all participants with values at these boundaries (*n* = 37 retained). This confirmed the robustness of the central findings: no significant differences were found between light conditions across all tasks (all p_Holm > 0.05). Clipping rates and full sensitivity analysis results are reported in Supplementary Appendix 3.

To assess whether the keypress artifact introduced bias into the pupillometric results, normalized pupil diameter variance was compared between affected and unaffected participants for each task-condition combination in which the artifact occurred, using independent-samples tests (Mann-Whitney U or independent *t*-test, depending on Shapiro-Wilk normality), with Holm correction for multiple comparisons. The artifact affected 11 participants (500 lux, 2-back), 12 (500 lux, 3-back), 14 (300 lux, 2-back), and 13 (300 lux, 3-back), out of 48. One of four comparisons (300 lux, 2-back) showed a nominally significant difference (*p* = 0.047), which did not survive Holm correction (p_Holm = 0.188); the remaining three comparisons were non-significant (all *p* ≥ 0.33).

### 3.3 Subjective measures

Regarding subjective measures, the only statistically significant difference between illuminances was observed for NASA-TLX scores during the 3-back task, which were higher at 500 lux than at 300 lux (*p* < 0.001, *d* = 0.81). No other significant differences were found for NASA-TLX in the remaining tasks, nor for any of the VAS Stress ratings across all tasks (all adjusted *p* > 0.05). Mean descriptive statistics for VAS ratings are presented in Table 4.

**Table 4:** Descriptive statistics for VAS scores by illuminance condition and task.

Condition	Task	Stress mean	Stress range	Fatigue mean	Fatigue range	Eye strain mean	Eye strain range
500 lux	2-back	36	0–80	40	0–90	44	0–100
	3-back	50	0–95				
	Writing	28	0–80				
300 lux	2-back	32	0–70	45	0–95	50	0–100
	3-back	50	0–95				
	Writing	25	0–74				

Fatigue and eye strain VAS were administered once per condition block (not per task), so per-task breakdown is not available for these measures. No statistically significant differences were found between illuminance conditions for fatigue or eye strain VAS scores.

Descriptive statistics for NASA-TLX ratings are presented in Table 5.

**Table 5:** Descriptive statistics for NASA-TLX scores by illuminance condition and task.

Condition	Task	Min	Max	Mean
500 lux	2-back	0	73	38
	3-back	16	85	57
	Writing	1	82	34
300 lux	2-back	6	85	36
	3-back	25	95	56
	Writing	0	81	32

Regarding perceived workload, a Friedman test on normalized data revealed a significant effect of task on all subjective scores: NASA-TLX for 500 lux (χ^2^ = 43.3, *p* < .001, Kendall's W: 0.451) and 300 lux (χ^2^ = 55.97, *p* < 0.001, Kendall's W: 0.583); VAS stress for 500 lux (χ^2^ = 32.35, *p* < 0.001, Kendall's W: 0.337) and 300 lux (χ^2^ = 50.66, *p* < 0.001, Kendall's W: 0.528).

Beyond the 3-back task, NASA-TLX and VAS scores did not differ significantly between illuminance conditions. Subjective responses across conditions were heterogeneous: 39% reported less stress under 500 lux; 50% reported less fatigue under 300 lux; 52% reported reduced eye strain under 500 lux; 40% reported lower workload under 500 lux.

### 3.4 Comparative sensitivity of measures

**Figure 4:**
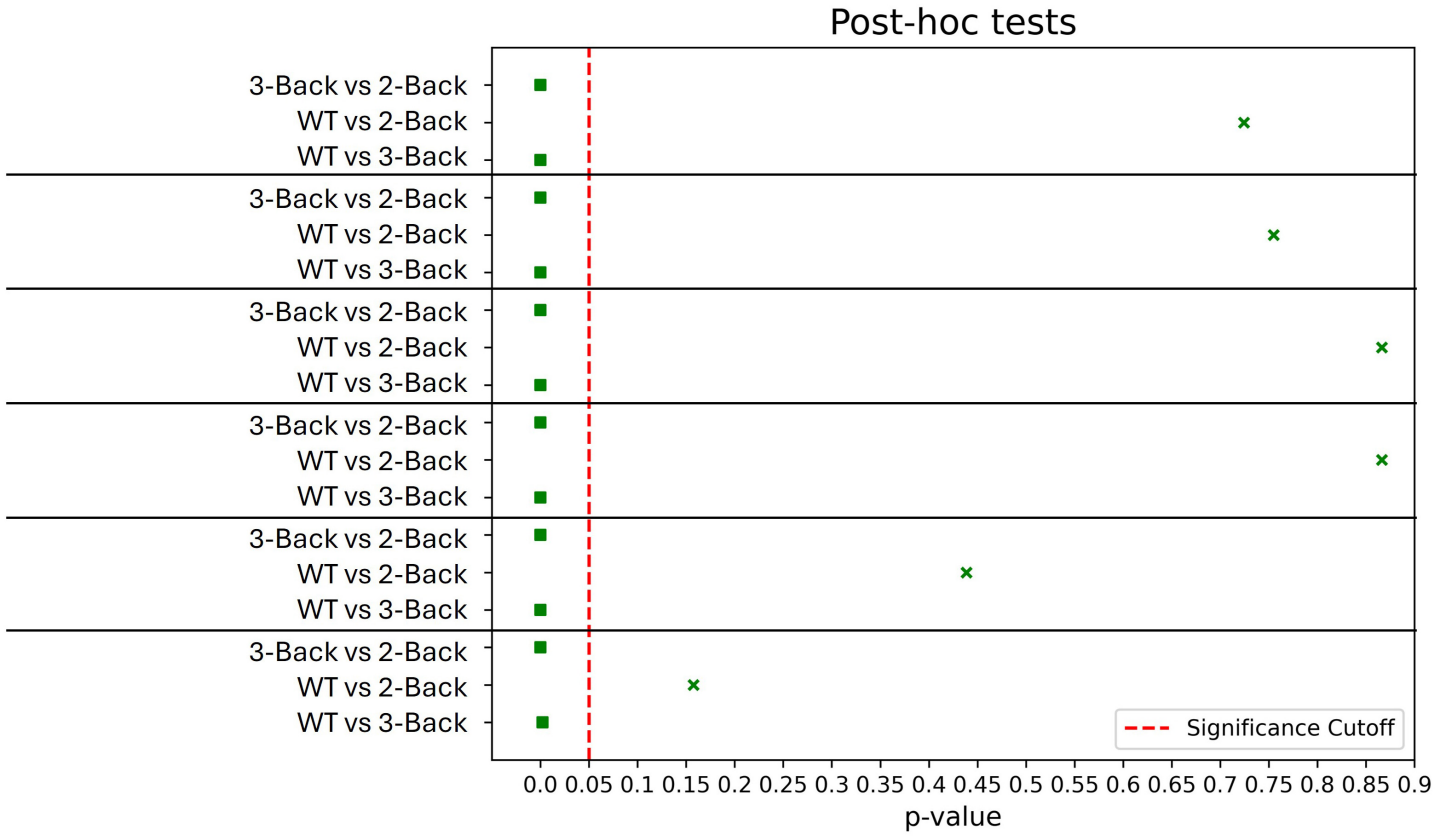
Graph showing *post-hoc p*-values from tests performed on pupil diameter variance, NASA-TLX scores, and stress VAS scores across both light conditions and all tasks. WT refers to the Writing task. Values not displayed on the graph have *p*-values greater than 0.7. Green squares represent significant values while green crosses represent non-significant values.

*Post-hoc* comparisons (Figure 4) revealed a clear symmetry in discriminative sensitivity between pupillometry and subjective measures. All tested measures in both conditions showed significant pairwise differences between the 3-back and 2-back tasks, and between the writing task and the 3-back task (all *p* < 0.05). The only non-significant comparison was writing task vs. 2-back (PD variance: *p* ≈ 0.16 at 500 lux; *p* ≈ 0.44 at 300 lux; NASA-TLX: *p* ≈ 0.75 at 500 lux; *p* ≈ 0.87 at 300 lux; VAS stress: p ≈ 0.72 at 500 lux; *p* ≈ 0.87 at 300 lux), likely reflecting the novelty-driven elevation of the 2-back response discussed in Section 4.3. Figure 4 presents *p*-values for all *post-hoc* comparisons; values above 0.7 are not displayed. Filled squares denote *p* < 0.05; crosses denote non-significant comparisons.

## 4 Discussion

This study investigated whether pupillometry could detect differences in cognitive load associated with two illuminance levels (300 and 500 lux) during screen-based tasks, and how its sensitivity compared to subjective self-report measures. The findings provide partial support for the value of pupillometry as a complementary occupational health assessment tool, while also highlighting important methodological constraints.

### 4.1 Illuminance and pupillary dynamics

Literature states that adequate illuminance reduces the cognitive load associated with visual processing, functioning as what has been described as an “environmental scaffold” that lowers perceptual burden (36, 37). However, in this experiment no significant differences were found between illuminance levels in pupillary metrics. Also, the 300 lux condition showing a higher constriction gradient (79.17% vs. 75% from first to last five seconds) is not statistically significant. It should be noted that this study did not measure screen surface luminance under each condition, which precludes full separation of ambient illuminance effects from potential contrast-mediated changes in task difficulty, a limitation that future studies should address.

Sensitivity analyses excluding pupil values at the plausibility filter boundary confirmed that the absence of significant illuminance-related differences in variance, amplitude, mean, and maximum pupil diameter was not attributable to sample size or boundary truncation effects.

### 4.2 Divergence between objective and subjective measures

Literature states that higher cognitive load elicits larger pupil diameter (38), and that low-urgency tasks are systematically underrated in subjective workload assessments relative to their actual physiological cost (39, 40). However, regarding task-level sensitivity both objective and subjective metrics detected significant pairwise differences between task demand levels consistently under both illuminance conditions. The writing task and 2-back task which were supposed to be the higher and lower demanding tasks, respectively, showed non-significant differences in all measures in both light conditions. These may be due to fixed task order.

Regarding illuminance sensitivity, only NASA-TLX in 3-back task was significantly perceived as larger at 500 lux then 300 lux (*p* < 0.001, *d* = 0.81). All other subjective and objective measures showed no significant differences between 300 and 500 lux in any task. These findings indicate that the two illuminance levels did not produce distinguishable effects on physiological pupil dynamics in this sample, despite the subjective perception of higher workload during the most demanding task under the higher illuminance condition. The absence of a consistent subjective preference for 500 lux on workload ratings, combined with 50% of participants reporting less fatigue under 300 lux, illustrates a pattern already identified in occupational health research: lower ambient stimulation may be perceived as more comfortable (41, 42). In summary, while the present study does not provide physiological evidence for a clear advantage of 500 lux over 300 lux in terms of pupil dynamics, it does highlight the importance of considering subjective responses when evaluating workplace lighting conditions. The dissociation between objective and subjective measures reinforces the central methodological argument: occupational health assessments of lighting adequacy should incorporate both types of indicators to capture the full spectrum of worker experience and physiological state.

From a practical standpoint, these findings suggest that increasing illuminance beyond 300 lux for predominantly screen-based, high-demand cognitive tasks may not yield a measurable physiological benefit and may even increase perceived workload without a corresponding reduction in objective strain. This supports flexible, task-specific illuminance guidance over a uniform 500-lux standard for all office work and suggests that worker-reported comfort and workload should inform lighting decisions alongside fixed illuminance thresholds, particularly for demanding, screen-intensive tasks.

### 4.3 Task order and novelty effects

An important interpretive constraint concerns the fixed task order (2-back → 3-back → writing). As acknowledged in the Methods, this design was not counterbalanced across participants. The elevated pupillary measures observed during the 2-back task relative to the 3-back task, despite the 3-back being more cognitively demanding, are consistent with a novelty effect: the initial, unfamiliar task elicits heightened physiological arousal that attenuates with exposure (43). For pupillometry specifically, habituation over time is an established phenomenon, and sequential task order means that condition effects on later tasks (3-back, writing) are partially confounded with practice and fatigue effects.

### 4.4 Limitations

A few limitations should be acknowledged. First, the fixed task order (2-back → 3-back → writing) was not counterbalanced. The habituation-driven reduction in pupillary responsiveness across the session cannot be fully separated from illuminance effects in the later tasks. Future protocols should implement a fully counterbalanced or randomized task sequence.

Second, screen contrast under each illuminance condition was not formally measured. Emissive displays reflect a portion of ambient light, which can reduce displayed contrast at higher ambient illuminance, a confound that has been discussed in the literature (15) and that cannot be excluded as a partial contributor to the observed differences.

Third, the absence of valid behavioral performance data (N-back accuracy was compromised by a keypress artifact) limits the study to physiological and subjective indicators. However, because participants received no real-time feedback indicating whether a keypress had registered during either the N-back or writing tasks, and the artifact was identified only during *post-hoc* analysis, frustration-driven pupillary arousal is an unlikely explanation for any effect of this artifact; this was further confirmed empirically, with no significant differences in pupil diameter variance between affected and unaffected participants after correction for multiple comparisons (Section 3.2).

Finally, the sample was predominantly young adults (mean age 24), limiting generalizability to older working populations, who exhibit different pupillary reactivity and cognitive load profiles (35). The correlational findings reported in the Results section should be interpreted as hypothesis-generating rather than confirmatory.

## 5 Conclusion

This study found no differences between 300 and 500 lux in pupillary metrics, while NASA-TLX revealed higher perceived workload at 500 lux during the most demanding task. Both objective and subjective measures showed task-level sensitivity though this should be interpreted alongside the fixed, non-counterbalanced task order.

These findings illustrate the methodological value of combining objective physiological metrics with subjective self-report: the two approaches capture different, and complementary, aspects of the worker's cognitive and experiential state. The divergence between them, rather than being a study limitation, represents a substantive finding with implications for how occupational health assessments of lighting adequacy to different tasks should be designed.

## 6 Future perspectives

Future research should counterbalance task order, profile screen luminance, and consider adding physiological measures such as EEG or heart rate variability to help triangulate cognitive state. A more age-diverse sample would extend the generalizability of findings to older working populations.

Investigating a broader range of illuminance levels (e.g., 400 and 750 lux) would clarify whether the observed effects follow a linear dose-response or a threshold pattern. Longer-term, developing adaptive lighting systems that respond in real time to pupillometric indicators of cognitive load is a promising avenue for smart workplace ergonomics. Validating this approach across diverse occupational settings would support evidence-based, personalized illuminance standards.

## Data Availability

The raw data supporting the conclusions of this article will be made available by the authors, without undue reservation.
