# Supplementary material for: Beyond visual comfort: comparing pupillometry and subjective measures of cognitive strain in workplace lighting
Source: Front Public Health. 2026 Sep 3;14:1875100. doi: 10.3389/fpubh.2026.1875100 (PMC13582193; doi:10.3389/fpubh.2026.1875100)
Supplement: Supplementary file 1 [file Supplementary_File_1.pdf]

Note: This document is best viewed and completed with Adobe Acrobat Reader.

ENLIGHT

Release 1.0.2, 16 October 2023

ENLIGHT Checklist

Below is the **ENLIGHT Checklist** for reporting ocular light exposures in human laboratory-based studies. We will strongly encourage that this checklist be used in conjunction with the **ENLIGHT Explanation & Elaboration (E&E) document**. This checklist is intended both to help authors, reviewers, and editors in evaluating the completeness of reporting in submitted studies, and for documentation of studies after publication. In the location column, please indicate the page, figure, or table number where the item or description can be found. If an item is not available, please select "**Not available**". If you consider an item not to be applicable in your specific study design after consulting the guidelines, please select "**Not applicable**". Items which do not have the option to select "Not applicable" were rated by experts as applicable for all studies, regardless of context. If you are unable to provide the information, please select "Not available".

The **ENLIGHT Checklist** (this document) and the **ENLIGHT E&E document** are released under the CC-BY-NC-ND License. For more information, please visit http://enlight-statement.org/.

General Information

Author names: D. Filipa Ferreira, Ana-Carolina Fonseca, Simão Ferreira, Luís Coelho, Matilde A. Rodrigu

Title of manuscript: Beyond Visual Comfort: Using Pupillometry to Objectively Assess Cognitive Performance F

Date:

A. Study Characteristics

A.1. Protocol-level characteristics

	Location (page, figure, table number)	Not available	Not applicable
Description of experimental setting	2.1 and 2.2	☐	
Timeline of experiment (including timing and duration of light)	2.4 and figure 2	☐	
Pre-laboratory sleep-wake/rest-activity behaviour		☐	☑
Pre-laboratory light exposure		☐	☑
Immediate prior light exposure (in laboratory)		☐	☑

A.2. Measurement-level characteristics

Measurement plane (e.g., horizontal or vertical)		☑	
Measurement viewpoint and location	2.2	☐	
Type, make and manufacturer of the measurement instrument	Table 1	☐	
Calibration status of the instrument		☑	☐

A.3. Participant-level characteristics

Ocular health and functioning	3.1	☐	
Pupil size and/or dilation	3.2	☐	☐
Relative time (e.g. to circadian phase or sleep)		☐	☑

B. Light characteristics

B.1. Light source type(s). Please select all that are relevant.

Room illumination (overhead or other)	Emissive surfaces including displays (incl. light therapy devices)	Wearable light emitting glasses	Ganzfeld exposure	Other:
☑	☐	☐	☐	☐

Polychromatic light	Monochromatic or narrowband light
☐	☐

	Location (page, figure, table number)	Not available	Not applicable
Type, make and manufacturer of the light source		☑	
Use of wearable filtering apparatus (e.g., blue-blocking glasses)		☑	☐

B.2. Light level characteristics

Illuminance (lux) and/or luminance (cd/m^2)	2.2	☐	
Spectral irradiance and/or radiance distribution		☐	☑
α−opic irradiance and/or radiance (including melanopic)		☐	☑
α−opic equivalent daylight illuminance and/or luminance (EDI/EDL, including melanopic)		☐	☑

NOTE: Luminance and radiance metrics (as opposed to illuminance and irradiance) are mainly relevant for emissive surfaces.

B.3. Colour characteristics

Peak wavelength and bandwidth	2.2	☐	☐
Colour appearance quantities (any)		☐	☑
Colour rendering metrics (any)		☐	☑

NOTE: Peak wavelength and bandwidth are most relevant for monochromatic or narrowband light sources.

B.4. Temporal and spatial characteristics

Location of stimulus and viewing distance	2.2	☐	
Temporal pattern (including flash frequency and waveform)	2.2	☐	☐
Relative or absolute size of the stimulus	2.2	☐	☐

Reset form to default values **Print as PDF**
