# Supplementary material for: Beyond visual comfort: comparing pupillometry and subjective measures of cognitive strain in workplace lighting
Source: Front Public Health. 2026 Sep 3;14:1875100. doi: 10.3389/fpubh.2026.1875100 (PMC13582193; doi:10.3389/fpubh.2026.1875100)
Supplement: Supplementary file 2 [file Supplementary_File_2.docx]

Appendix 2

Shapiro–Wilk test results for each normalized variable individually (per metric, condition, and task). These values were used to inform the choice between Pearson and Spearman correlation coefficients. FP – Problem-Focused Coping; EP - Emotion-Focused Coping; EC – Avoidant Coping; Score_X: S – Stress (DASS-21), A – Anxiety (DASS-21), D-Depression (DASS-21), W- Worry (PSWQ-16); PD – Pupil diameter; Max – Maximum; Min – Minimum; Amp – Amplitude; Var – Variation. WT – Writing Task.

|  | | Variable | Statistic value | p-value | Normal? (p>0.05) |
| --- | --- | --- | --- | --- | --- |
| Initial Questionnaire | | Age | 0.838 | 0.000 | False |
|  |  | FP | 0.950 | 0.040 | False |
|  |  | EP | 0.872 | 0.000 | False |
|  |  | EC | 0.818 | 0.000 | False |
|  |  | Score_S | 0.976 | 0.438 | True |
|  |  | Score_A | 0.960 | 0.101 | True |
|  |  | Score_D | 0.934 | 0.009 | False |
|  |  | Score_W | 0.972 | 0.308 | True |
| 500 lux | 2-Back | NASA-TLX | 0.976 | 0.424 | True |
|  |  | Max PD | 0.905 | 0.001 | False |
|  |  | Min PD | 0.902 | 0.001 | False |
|  |  | Amp PD | 0.956 | 0.068 | True |
|  |  | Mean PD | 0.981 | 0.625 | True |
|  |  | Var PD | 0.868 | 0.000 | False |
|  | 3-Back | NASA-TLX | 0.947 | 0.031 | False |
|  |  | Max PD | 0.988 | 0.885 | True |
|  |  | Min PD | 0.917 | 0.002 | False |
|  |  | Amp PD | 0.968 | 0.221 | True |
|  |  | Mean PD | 0.983 | 0.697 | True |
|  |  | Var PD | 0.727 | 0.000 | False |
|  | WT | NASA-TLX | 0.960 | 0.105 | True |
|  |  | Max PD | 0.966 | 0.176 | True |
|  |  | Min PD | 0.900 | 0.001 | False |
|  |  | Amp PD | 0.973 | 0.326 | True |
|  |  | Mean PD | 0.965 | 0.168 | True |
|  |  | Var PD | 0.884 | 0.000 | False |
|  | VAS_S | 2-Back | 0.967 | 0.193 | True |
|  |  | 3-Back | 0.838 | 0.000 | False |
|  |  | WT | 0.917 | 0.002 | False |
| 300 lux | 2-Back | NASA-TLX | 0.962 | 0.121 | True |
|  |  | Max PD | 0.982 | 0.664 | True |
|  |  | Min PD | 0.933 | 0.009 | False |
|  |  | Amp PD | 0.952 | 0.047 | False |
|  |  | Mean PD | 0.980 | 0.573 | True |
|  |  | Var PD | 0.872 | 0.000 | False |
|  | 3-Back | NASA-TLX | 0.967 | 0.192 | True |
|  |  | Max PD | 0.986 | 0.839 | True |
|  |  | Min PD | 0.646 | 0.000 | False |
|  |  | Amp PD | 0.946 | 0.029 | False |
|  |  | Mean PD | 0.958 | 0.080 | True |
|  |  | Var PD | 0.877 | 0.000 | False |
|  | WT | NASA-TLX | 0.982 | 0.665 | True |
|  |  | Max PD | 0.964 | 0.143 | True |
|  |  | Min PD | 0.924 | 0.004 | False |
|  |  | Amp PD | 0.908 | 0.001 | False |
|  |  | Mean PD | 0.939 | 0.015 | False |
|  |  | Var PD | 0.877 | 0.000 | False |
|  | VAS_S | 2-Back | 0.920 | 0.003 | False |
|  |  | 3-Back | 0.957 | 0.078 | True |
|  |  | WT | 0.843 | 0.000 | False |
