# Supplementary material for: Beyond visual comfort: comparing pupillometry and subjective measures of cognitive strain in workplace lighting
Source: Front Public Health. 2026 Sep 3;14:1875100. doi: 10.3389/fpubh.2026.1875100 (PMC13582193; doi:10.3389/fpubh.2026.1875100)
Supplement: Supplementary file 3 [file Supplementary_File_3.docx]

Appendix 3

A: Clipping/truncation rates (% of participants with a pupil value at the 2–8 mm filter boundary):

| **Task** | **500 lux** | **300 lux** |
| --- | --- | --- |
| 2-back | 6.25% | 8.33% |
| 3-back | 8.33% | 4.17% |
| Writing task | 2.08% | 6.25% |

B: Sensitivity analysis excluding boundary cases (PD – pupil diameter):

| Metric | Task | Test | p (uncorrected) | p (Holm) | Effect size |
| --- | --- | --- | --- | --- | --- |
| PD variance | 2-back | paired t-test | 0.2014 | 0.5284 | -0.2139 |
|  | 3-back | Wilcoxon | 0.4372 | 0.5284 | 0.1277 |
|  | Writing | paired t-test | 0.1761 | 0.5284 | -0.2269 |
| PD amplitude | 2-back | paired t-test | 0.4546 | 0.5134 | -0.1243 |
|  | 3-back | Wilcoxon | 0.2364 | 0.5134 | 0.1947 |
|  | Writing | paired t-test | 0.1711 | 0.5134 | -0.2296 |
| PD mean | 2-back | paired t-test | 0.9472 | 1.0000 | -0.0110 |
|  | 3-back | paired t-test | 0.6894 | 1.0000 | -0.0662 |
|  | Writing | paired t-test | 0.9147 | 1.0000 | -0.0177 |
| PD maximum | 2-back | paired t-test | 0.6000 | 1.0000 | -0.0870 |
|  | 3-back | paired t-test | 0.6159 | 1.0000 | -0.0832 |
|  | Writing | paired t-test | 0.4639 | 1.0000 | -0.1217 |
| PD minimum | 2-back | paired t-test | 0.5348 | 0.9965 | 0.1030 |
|  | 3-back | paired t-test | 0.3322 | 0.9965 | 0.1616 |
|  | Writing | paired t-test | 0.4347 | 0.9965 | 0.1299 |
